# Trainees’ views of physician workforce policy in Quebec and their impact on career intentions

**Published:** 2014-12-17

**Authors:** Julie Hallet, Nathalie Saad, Mathieu Rousseau, François Lauzier

**Affiliations:** 1 Departments of Surgery and Social & Preventive Medicine, Université Laval, Quebec City, Quebec; 2 Centre de Recherche du CHU de Québec, Traumatologie-Urgence-Soins Intensifs, Departments of Medicine and Anesthesiology, Division of Critical Care Medicine, Université Laval, Quebec City, Quebec; 3 Department of Medicine, McGill University, Montreal, Quebec; 4 Fédération des Médecins Résidents du Québec (FMRQ), Montreal, Quebec; 5 Department of Surgery, McGill University, Montreal, Quebec

## Abstract

**Background:**

The physician workforce in Quebec is regulated by a government-controlled plan. Many specialty trainees expressed concerns about securing a position. Our objective was to analyze physicians’ employment issues in Quebec and their impact on residents’ training in specialty programs.

**Methods:**

We distributed a web-based self-administrated survey to all Quebec residents training in specialty programs to capture data about residents’ ability to find employment, career plans and perceptions regarding the workforce policy. Three groups were considered: graduates, non-graduating senior residents, and junior residents.

**Results:**

The overall response rate was 41.5% (985/2372). 47.3% of graduates did not have a position two months before finishing their training. Among residents without a position, 27.1% of graduates intend to leave Quebec, and 19.6% to complete a fellowship to postpone their start in practice. Overall, 77.9% of respondents believed there are not enough job opportunities for the number of trainees.

**Conclusion:**

Quebec specialty residents experience significant difficulties obtaining a position in the province and perceive that there are not enough job opportunities, which impacts their career plans and could drive them to complete a fellowship or plan to practice outside the province. Trainees’ experience in finding employment needs to be considered in planning the physician workforce.

## Introduction

Perceived physician shortage in Quebec has resulted in the development and application of strict governmental policies around workforce development, especially in specialty medicine.^1^ The number of residents in training was increased by 49.3% from 2154 in 2000 to 3215 in 2012 (*Conférence des Recteurs et des Principaux des Universités du Québec* [CREPUQ], Marie Galibois, personal communication, May 2012). During the same period, the Ministry of Health and Social Services (MSSS) restricted the existent recruitment plan for physicians in each of the administrative regions in Quebec, known as the regional medical staffing plan (*Plan Régionaux d’Effectifs Médicaux*), in an attempt to ensure an adequate and equitable distribution of physicians in all regions of the province.^1^ In 2010, the MSSS presented a five-year plan aimed at planning physician distribution in the province from 2011 to 2015.^1^ Following the announcement of this plan, trainees have voiced their concerns about finding an adequate position, as the government-planned job opportunities for graduating specialists appeared sparse and inflexible.^2^

Previous studies have addressed the physician workforce organization in Canada by specialty. For some, such as neurosurgery, cardiac surgery or radio-oncology, concern of an oversupply of physicians in Canada has already become reality.^3^–^6^ However, there are no data on the impact of provincial planning on residents’ perceptions of job opportunities and on how it affects transition into practice. Our goal was to describe this perspective, given that the first evaluation of the five-year plan was scheduled at year 3 in 2012. We performed a province-wide survey of residents training in specialty programs to define residents’ ability to find employment, future career plans and perceptions regarding the workforce policy.

## Materials and Methods

**Figure 1:**
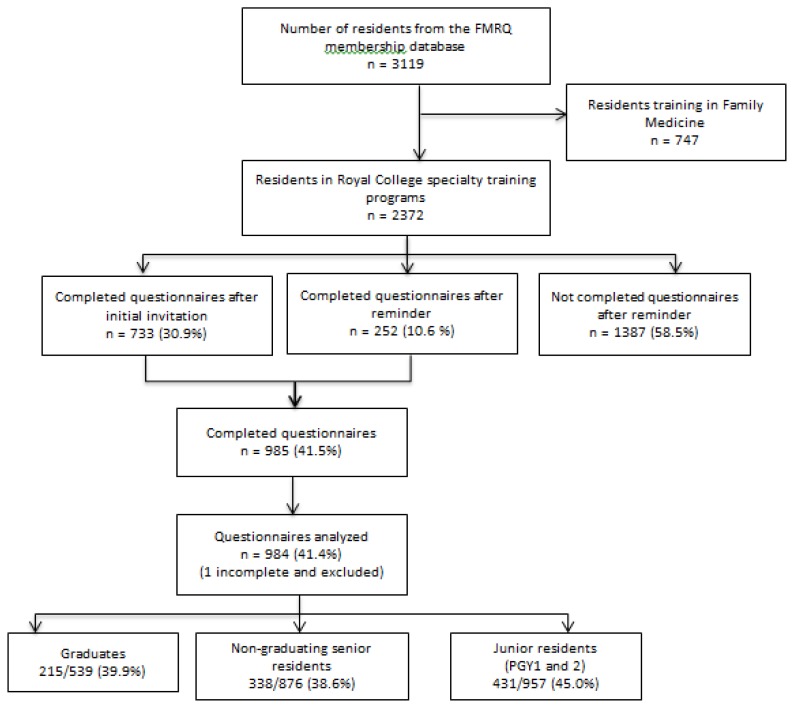
Flow diagram of respondents

Under the auspices of the *Fédération des Médecins Résidents du Québec* (FMRQ), the union representing residents in Quebec, we conducted a self-administrated survey of all residents training in Royal College specialty programs in Quebec. Residents training in Family Medicine programs were excluded from the FMRQ database. Email addresses from potential respondents were obtained in April 2012 through the membership database of the FMRQ membership of which is mandatory. The final list included 2372 potential respondents, 539 of which were graduates who were completing their last year of training (Figure 1).

### Questionnaire development

The questionnaire was developed by an expert group that identified important domains and specific issues within those domains, highlighting those most pertinent to job opportunities for residents. We first generated items without restriction, grouped them in domains, and proceeded to item reduction to retain only the most relevant ones.^7^ The chosen domains were: (a) ability to find employment, (b) future career plans, (c) the application process for a position and (d) perception of the current five-year plan. We used a five-point Likert scale to assess the perceptions of respondents and a 0 to 10-point scale to evaluate stress related to job searching.

To assess the clarity and interpretation of the questionnaire, we pre-tested it with specialists who had recently graduated. The expert group evaluated face validity, clarity and comprehensiveness through a clinical sensibility analysis.^7^ The original French and English questionnaires were test-piloted by 5 graduate residents from different training programs; those residents were part of the final population to whom the survey was administered. Residents were asked to provide feedback about the flow, the clarity and the ease of administration of the questionnaire. The questionnaire was revised accordingly. The final questionnaire is available in Appendix 1.

### Questionnaire administration

The survey was self-administered in both English and French using web-based software (Fluidsurveys: www.fluidsurveys.com, Chide.it Inc., Ottawa, Canada) in May 2012. Each potential respondent received an individual invitation from the FMRQ to complete the survey. An electronic reminder to non-responders was sent after 10 days. No incentive was offered to complete the survey.

Since junior residents are not yet involved in a job searching process and the current workforce policy provides a list of available positions up until 2015, questions regarding the ability to find employment and future career plans were directed towards senior residents only (graduates and non-graduating senior residents). Other general questions were addressed to all residents.

### Data analysis

Considering the response rate obtained in previous similar specialty-based surveys, we expected a response rate of 50% (1186 respondents).^5^,^8^ Using a 95% confidence interval, this sample size provides a 3.1% precision for answers to categorical questions based on a conservative estimate that the true proportion is 50%. We first performed a descriptive analysis of completed questionnaires. As all survey questions captured categorical data, we report responses with proportions (n/N) and a 95% confidence interval (95% CI). We included data from incomplete questionnaires. The denominators for certain analyses were adjusted accordingly. We divided the respondents into several groups: graduates (residents in their final year of training) and non-graduating senior residents (post-graduate year [PGY] 3 and above but not in the final year of residency), and junior residents (PGY 1 and 2). We conducted a comparative analysis of responses of graduates and non-graduating senior residents who did not secure a position, to explore changes in plans with the progression of residency training. To this end, we performed a univariate analysis comparing response rates and answers from these groups, using the Fisher’s exact test or the Pearson Chi square test when appropriate for categorical data and the Kruskal-Wallis test for ordinal data. Data were considered statistically significant at *p* ≤ 0.05. Statistical analyses were conducted using XLSTAT version 2011.5 (Addinsoft SARL, Paris, France) for Microsoft Excel. Answers to open-ended questions were coded independently by two of the authors (NS and JH), in both languages, and classification was performed by consensus. Results are reported grouped in themes and dimensions within each theme.

### Funding and ethical issues

The authors initially conducted this survey under the auspices of the FMRQ to elaborate a position statement on the regional medical staffing plan. No personal identifying data were collected. According to the Tri-Council Policy Statement,^9^ studies performed by organizations to evaluate their members according to their mandate do not need Research Ethics Board approval, as long as no identifiable data are used. Our study fulfilled those criteria and Research Ethics Board approval was not sought. However, we asked study participants for their consent (see Appendix 2).

## Results

### Characteristics of respondents

Overall, 41.5% of residents (985/2372) completed the questionnaire. The response rate did not differ among graduates (215/539, 39.9%), non-graduating senior residents (338/876, 38.6%) and junior residents (431/957, 45.0%) (*p* = 0.17). One respondent was excluded for having responded only to the training program question (Figure 1).

### Ability to find employment

These questions were specifically directed to graduates and to non-graduating senior residents, as the current five-year plan applies directly to them. Two months before the end of the academic year, 90.0% (296/329, 95% CI: 86.6–93.2%) of non-graduating senior residents, and 47.3% (99/209, 95% CI: 40.5–54.1%) of graduates did not have a hospital-based position (*p* < 0.01). Among residents without a position, 44.2% (122/276, 95% CI: 38.3–50.1%) of non-graduating senior residents and 79.8% (75/94, 95% CI: 71.7–87.9%) of graduates were actively negotiating for one (*p* < 0.01).

### Future career plans

**Figure 2:**
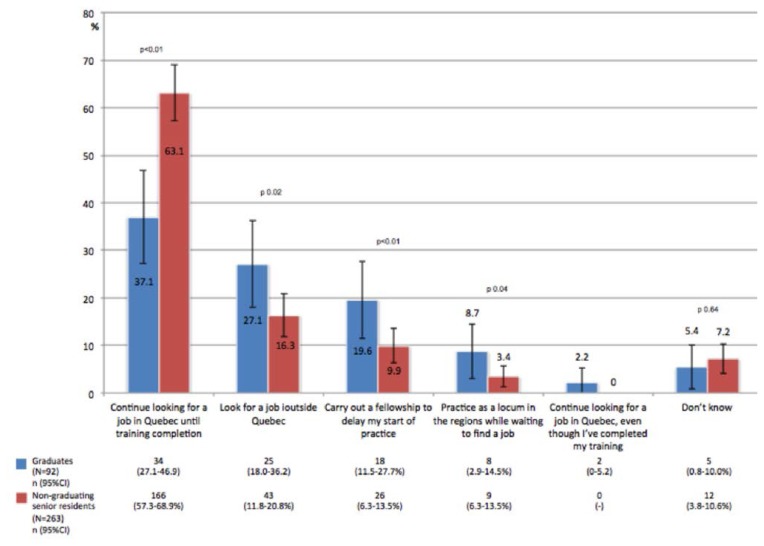
Comparison of plans of graduates and non-graduating senior residents without a position

The proportion of residents planning to pursue fellowship training did not differ between non-graduating senior residents (53.8%, 177/329, 95%CI: 48.8–58.8%) and graduates (54.2%, 115/212, 95%CI: 47.5–60.9%) (*p* = 0.92). Among senior residents without a position (Figure 2), graduating residents, compared to non-graduating senior residents, were less likely to continue searching for a job in Quebec (37.0% vs. 63.1%; p < 0.01), and were more likely to look for employment outside the province (27.1% vs. 16.3%; *p* = 0.02), to carry out a fellowship for postponing their entry into practice (19.6% vs. 9.9%; *p* < 0.01) and to practice as a locum physician while waiting to find employment (8.7% vs. 3.4%; *p* = 0.04).

When excluding respondents with a confirmed position (52.7% of graduates and 10.0% of non-graduating senior residents), 69.7% of graduates (62/89, 95% CI: 60.2–79.2%), 73.3% of non-graduating senior residents (187/255, 95% CI: 67.9–78.7%) and 79.0% of junior residents (290/367, 95% CI: 74.8–83.2%) were not confident in obtaining a position at the hospital of their choice (*p* = 0.09 for comparison among all groups). 50.0% of graduates (44/88, 95% CI: 39.6–60.4%), 40.6% of non-graduating senior residents (104/256, 95% CI: 34.6–46.6%), and 39.9% of junior residents (146/366, 95% CI: 34.9–44.9%) were not confident in securing a position in Quebec at all (*p* = 0.21). When only including respondents with a confirmed position, 21.8% of all graduates (43/197, 95%CI: 16.0–27.6) and 30.3% (90/297, 95%CI: 25.1–35.5) of non-graduating senior residents had considered finding a job outside Quebec during their training (*p* = 0.04).

### Perception of the current five-year plan

**Figure 3:**
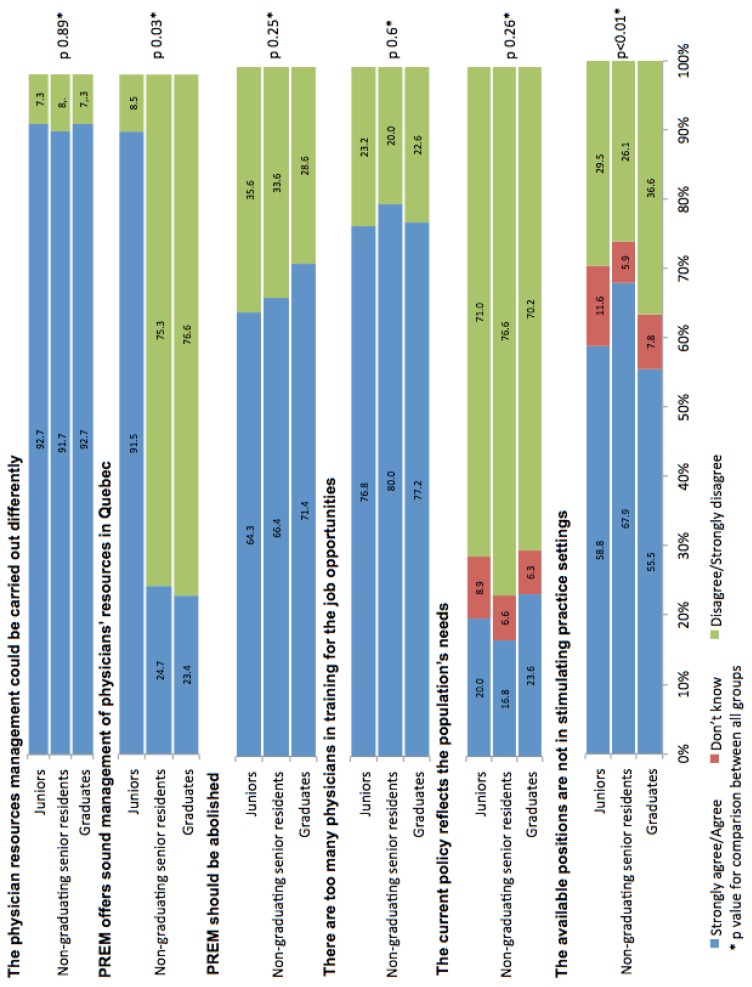
Comparison of residents’ perceptions of the current five-year plan for the physician workforce in Quebec, between graduates, non-graduating senior residents and junior residents

For this section, questions were directed to all residents. Regarding the efficiency of the current plan, 91.9% (776/844, 95% CI: 90.1–93.7%) of all respondents strongly agree or agree that physician resources management could be carried out differently. Furthermore, 72.2% (605/838, 95% CI: 69.2–75.2%) disagree or strongly disagree that the regional medical staffing plan offers sound management of physicians’ resources in Quebec, and 66.7% (554/830, 95% CI: 63.5–69.9%) believe that the regional medical staffing plan should be abolished. Most residents (77.9%, 654/840, 95% CI: 75.1–80.7%) agree or strongly agree that there are too many physicians in training for the number of job opportunities. A majority of residents believe that the available positions are not in stimulating practice settings (61.2%, 518/846, 95% CI: 57.9–64.5%). About the current distribution of positions in the province, most of the residents disagree or strongly disagree that the current policy reflects the population’s needs (72.9%, 616/845, 95% CI: 69.9–75.9%). Details on the perceptions of the current five-year plan according to each study group are provided in (Figure 3). There was no statistically significant difference between study groups, except for the statement that the regional medical staffing plan offers sound management of physicians’ resources (*p* = 0.03) and that available positions are not in stimulating practice settings (*p* < 0.01).

When asked to grade stress related to job searching on a 0 to 10 scale, the median score was 8 (interquartile range:7–9) among graduates, 8 (IQR:7–9) for non-graduating senior residents and 7 (IQR:6–8) for junior residents (*p* < 0.01 for comparison between study groups).

### Analysis of open-ended questions

Four themes regarding current workforce management emerged from our analysis. They are summarized below and outlined in Table 1.

#### Knowledge about the application process

Residents note that the process of obtaining a position lacks standardization and is complicated by administrative barriers. This results in a time-consuming process that takes more than a year to complete and represents a significant source of stress. This is true throughout all post-graduate years: “*I am only R2, but obtaining a position in a stimulating clinical setting is an important source of stress and frustration.*”

#### Inefficiency of positions management policy

Residents report that job positions are not properly matched to other healthcare resources allocation. For residents, position attribution appears unevenly distributed, resulting in a decentralization of services, as noted by one resident *“there seems to be a complete lack of prevision, especially regarding operating rooms, the establishments will not be able to let so many surgeons work”.*

#### Lack of positions

Globally, residents observe a lack of positions. They fear that authorities continue to admit residents to training despite already insufficient job opportunities. One resident noted: “*The programs continue to accept new residents in Quebec knowing they won’t be able to work here.”* Thus, despite wanting to work in Quebec, they feel forced to go abroad. Two residents mentioned: “*I would like to stay in Quebec to practice, particularly at a university centre, however due to the lack of PREM in my specialty, I am now looking outside of Quebec.”* and “*If there is no PREM, I might consider leaving Quebec even though I wish to stay here.*”

#### Lack of information

Residents noted that there is a lack of guidance at the hospital and governmental levels. Information is not passed on efficiently. Data available on the MHSS website are not up to date. One resident noted: *“I find this system to be quite confusing and misleading. We are told about the published lists on the MSSS website but reality does not seem to reflect this list.”*

## Discussion

This survey details the experience of residents in a medical specialty looking for a position in the province of Quebec. Despite residents actively negotiating for open government-controlled positions, almost half of graduates with less than 2 months before the end of their training and 90% of non-graduating senior residents to whom the current workforce plan applies are without a confirmed position. Among residents who intend to carry out a fellowship, approximately a quarter indicate the lack of a position as one of the reasons why they decided to prolong their training. More than three quarters of residents perceive that there are too many trainees for the number of job opportunities and that the available positions do not reflect the population’s needs. A very significant proportion of residents believe that sound management of the physician workforce could be carried out differently. Finally, one in four graduates has been considering working outside the province.

Previous data reported by Canadian medical societies identified the same issues. In 2006, an analysis of the Neurosurgery workforce in Canada concluded that the number of neurosurgeons currently trained is twice the number needed.^4^ In 2007, a national survey of Radiation Oncology graduates reported that fewer than 20% felt that there was a strong demand for Radiation Oncologists in Canada.^5^ Indeed, a survey of Radiation Oncologists graduating between 2000 and 2010 reported that although most respondents secured a position in Canada, many extended their training with a fellowship, and the potential difficulty for new graduates to find positions was a source of anxiety.^10^ Preliminary data from a national survey of residents writing their certification exams conducted by the *Royal College of Physicians and Surgeons of Canada (RCPSC)*, presented at the *Canadian Medical Association’s Specialist Forum* in September 2011, are raising similar concerns regarding the ability of Canadian residents to find employment. This survey had identified seven specialities experiencing difficulties with employment in Canada: Cardiac Surgery, Neurosurgery, Plastic Surgery, Nephrology, Public Health and Preventive Medicine, Otolaryngology and Radiation Oncology.^11^,^12^ In a recent message from its chief executing officer, the RCPSC reported that 14% of 2011 Canadian specialty graduates did not find a position within 12 to 14 weeks of their certification.^13^ Our data confirm these issues, but this is the first time that the extent of the problem is documented with a province-wide survey including all medical and surgical specialities. Contributing to the imbalance in job opportunities is the steady increase of individuals entering residency programs (CREPUQ, Marie Galibois, personal communication, May 2012). In the *Future of Medical Education in Canada – Post-Graduate* (FMEC-PG) project, the *Association of Faculties of Medicine of Canada*, the RCPSC, the *College of Family Physicians of Canada* and the *Collège des Médecins du Québec* already warned the Canadian and provincial authorities of the need to reassess residency position distribution.^14^ The 5-year plan intends to allow for a position for each resident in training and adds a 15% margin.^1^ For some specialities, such as Cardiology, Respirology, Nephrology, Cardiac Surgery, Neurosurgery, General Surgery and Orthopedic Surgery, this margin is not achieved,^1^ which may contribute to the perceived lack of job opportunities.

We observed that career planning is still a major stressor for residents and that job uncertainty modifies their career plans. More than half of residents intend to carry out a fellowship, and a fifth are doing so with the hope of obtaining a job. This phenomenon has already been reported among specific training programs. In 2009, most Canadian Radiation Oncology residents expressed plans to complete a fellowship, even if they believe their program adequately trains residents to enter practice.^5^ Data gathered by the CREPUQ reveals that 37% more graduates in Quebec are carrying out a fellowship in 2012 compared to 2002 (CREPUQ, Marie Galibois, personal communication, May 2012). Our results suggest that this phenomenon is likely to intensify. Among physicians who completed training in 2009 in Quebec, 83.5% were practicing in Quebec in 2011.^15^ The future career plans that residents expressed in this survey could change these numbers. Indeed, more than a quarter of graduates without a position plan to look for a position outside of Quebec.

Our study has several limitations. The 41.5% response rate suggests caution in considering the conclusions from this survey, as do the variation of the denominators due to incomplete questionnaires. The use of incentives could have helped in increasing this response rate. We also recognize that many questions examined stated plans, which may not be fully understood and may change over time. However, when considering the best-case scenario in which all non-respondents have confirmed positions, our results are still worrisome since at least 99/539 (18.3%) graduating residents and 296/876 (33.8%) non-graduating senior residents would be without a position. Our study does not provide data broken down by training program and we realize that the job reality for each specialty varies. Demographic changes, increase in chronic diseases, variation within a population, changes in the contemporary clinical practice, technological advances, sub-specialization and new academic needs have to be accounted for.^4^,^16^,^17^ Also, we did not look at data according to the university affiliations, age, sex, and nationality in order to ensure the anonymity of the respondents.

We acknowledge the difficulty for stakeholders to foresee healthcare needs and fulfil societal needs. Our goal was to depict the impacts of the macro-management of a comprehensive workforce planning policy that was designed through a population-based rather than a specialty-based approach. Our results highlight that physician workforce planning represents a major challenge for all stakeholders, including trainees. The balance between the number of trainees in medical school and residency programs, and societal needs for healthcare can be fragile and tentative. The Quebec experience depicted here from the perspective of residents, seems to indicate that a very authoritarian approach to physician workforce planning could have unforeseen consequences, such as modification of career plans (fellowships) or medical migration.

## Conclusion

In our study, we observed that, given the current restricted governmental policies, residents training in specialty programs in Quebec report difficulties in obtaining a hospital-based position in the province. They believe there are not enough job opportunities for the number of residents in training. This situation impacts on their career plans, driving them to carry out a fellowship or plan to practice outside of Quebec. In the setting of a single government employer for physicians, trainees’ experiences in finding employment has to be considered in planning the physician workforce. Further research is needed to determine the actual availability of positions in Quebec, and the path of graduates over time, in order to review and adapt the current physician workforce management accordingly.

## Figures and Tables

**Table 1:** Themes and dimensions of residents’ perceptions of current physicians positions’ management in Quebec

Theme	Dimensions
Complexity of the application process	Lack of standardization Lack of direction on how to obtain a position Multiplicity of administrative barriers Additional source of stress Time-consuming process
Inefficiency of the positions management policy	Job offers despite no official positions in the MHSS plan Mismatch between physicians positions and resource allocations Detrimental decentralization of services Recruitments of IMGs
Lack of positions	Mismatch between the number of graduates, retirements and new positions Positions do not reflect the expressed needs of departments Misrepresentation of needs in positions Virtual positions Obligation to practise outside of Quebec
Lack of information	Lack of guidance at the hospital level Lack of guidance at the governmental level Autocratic decisional process Misleading data available from the authorities (MHSS website)

MHSS: Ministry of Health and Social Services; IMG: International Medical Graduates

## Appendix 1. Questionnaire

**This survey will be used to evaluate the situation with respect to PREMs in Quebec and to draw up a profile of medical residents’ opinions concerning PREMs. In this way, we will be able to plan our action in this regard more effectively, and to represent you better.**

Rest assured that all information provided will be treated anonymously.

**Which residency program are you currently registered in?**

**What is your residency level?**

Questions directed to all respondents.

○ R1
○ R2
○ R3
○ R4
○ R5
○ R6
○ R7 and above

**Do you plan to perform a fellowship before starting out in practice?**

Questions directed to PGY-3 and more.

○ Yes
○ No
○ I am currently completing a fellowship
○ I don’t know

**Do you currently have a position confirmed in an establishment’s PEM?**

Questions directed to PGY-3 and more.

○ Yes
○ No

**NO CONFIRMED PREM**

Questions directed to respondents answering “no” to the question “Do you currently have a position confirmed in a establishment PEM?”.

**If you do not have a PREM, are you currently negotiating for one with an establishment?**

○ Yes
○ No
○ I will not be practicing in Quebec

**If you do not currently have a PREM, what are the main reasons for this? Rank the following potential reasons by order of importance:**

Waiting for results from applications initiated

○ 1
○ 2
○ 3
○ 4

Too few available positions in my specialty

○ 1
○ 2
○ 3
○ 4

Lack of positions that suit me in my specialty

○ 1
○ 2
○ 3
○ 4

Other

○ 1
○ 2
○ 3
○ 4

**If you answered “Other” to the previous question, please specify:**

**If you do not currently have a PREM, indicate what best represents what you are planning to do now?**

○ Continue looking for a PREM in Quebec, until my training is completed
○ Continue looking for a PREM in Quebec, even though I’ve completed my training
○ Practice as a locum in the regions while waiting to find a PREM
○ Carry out a fellowship to delay my start of practice
○ Look for a position in my specialty outside Quebec
○ Don’t know

**GENERAL VIEWS CONCERNING PREMs**

Questions directed to all respondents.

**To what extent do the following statements reflect your views on PREMs?**

Indicate one choice per line.

**Table:**

	Completely agree	Agree	Disagree	Completely disagree
PREMs lead to sound management of physician resources in Quebec.	○	○	○	○
Physician resource management in Quebec could be carried out otherwise than through PREMs.	○	○	○	○
PREMs should be abolished.	○	○	○	○
There are too many physicians in training for the recruitment opportunities available.	○	○	○	○

**In your specialty, indicate to what extent you agree with the following statements:**

**Table:**

	Completely agree	Agree	Disagree	Completely disagree
There are enough PREMs available for residents in training.	○	○	○	○
There are enough PREMs in university settings to meet needs.	○	○	○	○
The distribution of PREMs reflects the needs of the population.	○	○	○	○
The available PREMs are not in stimulating practice settings.	○	○	○	○
I am confident of obtaining a position in Quebec.	○	○	○	○

**I am confident in obtaining a position in the establishment of my choice.**

○ Yes
○ No
○ Don’t know

**Are you planning to practice outside Quebec?**

○ Yes
○ No
○ Don’t know

**THE PREM PROCESS**

Questions directed to all respondents.

**On a scale from 0 a 10 (from not at all stressful to highly stressful), how much of a source of stress does looking for a PREM represent for you?**

○ 0
○ 1
○ 2
○ 3
○ 4
○ 5
○ 6
○ 7
○ 8
○ 9
○ 10
